# Chronic cervical instability in mice and rats: a reproducible model to simulate human intervertebral disc degeneration

**DOI:** 10.7717/peerj.20465

**Published:** 2025-12-08

**Authors:** Kewu Tu, Junhao Tan, Guanhai Zeng, Fuzhou Xu, Dongteng Liao, Weiqi Lu, Kun Zhao, Zhaomou Chen, Beidi Zhou, Xiangheng Dai

**Affiliations:** 1 Department of Spinal Surgery, Shaoguan First People’s Hospital, Affiliated Hospital of Shaoguan University, Shaoguan, Guangdong, China; 2 Department of Osteoarthritis, Yuebei People’s Hospital Affiliated to Shantou University Medical College, Shaoguan, Guangdong, China; 3 Division of Spine Surgery, Department of Orthopaedics, Nanfang Hospital, Southern Medical University, Guangzhou, Guangdong, China; 4 The First Clinical College of Guangdong Medical University, Zhanjiang, Guangdong, China; 5 Department of Prevention and Health Care, Shaoguan First People’s Hospital, Affiliated Hospital of Shaoguan University, Shaoguan, Guangdong, China

**Keywords:** Intervertebral disc degeneration, Cervical instability, Mouse model, MRI analysis, Histological evaluation, Rat model

## Abstract

**Background:**

Chronic low-back pain, a leading cause of global disability, is closely linked to intervertebral disc degeneration (IVDD). Traditional animal models have faced challenges in replicating the gradual, chronic nature of human IVDD.

**Objective:**

To address these limitations, we aimed to develop a novel mouse model of cervical spine instability that more accurately mimics the progressive degeneration observed in humans.

**Animals:**

This study used 48 male Sprague–Dawley rats (3 months old, weighing 230 ± 20 g) and 48 male C57BL/6 mice (12 weeks old, weighing 20 ± 2 g).

**Methods:**

A bilateral cervical laminectomy combined with spinous process resection was performed while preserving the facet joints and posterior cervical muscles to induce chronic intervertebral instability. Longitudinal assessments were conducted using *in vivo* magnetic resonance imaging (MRI), histological staining (H&E and Safranin O-fast green), immunofluorescence and western blot analyses at 4, 8, and 12 weeks post-operation.

**Results:**

MRI findings demonstrated progressive degeneration at the C4/5, C5/6, and C6/7 levels, with the most pronounced changes observed at 4 and 8 weeks post-surgery and partial recovery at 12 weeks. H&E and Safranin O staining confirmed significant cellular loss, structural disorganization, and proteoglycan depletion in the affected discs. Immunofluorescence staining revealed a progressive decrease in collagen type II and aggrecan expression over time. Conversely, collagen type I expression increased, indicating a shift toward fibrosis. Western blot analysis confirmed elevated levels of oxidative stress markers (albumin and AOPPs), matrix metalloproteinases (MMP3 and MMP13), senescence markers (p53, p21, p16), and inflammatory cytokines (IL-1β, TNF-α) at 4 and 8 weeks, with a partial decline by 12 weeks.

**Conclusions:**

This innovative cervical instability model not only minimizes the risk of nerve injury and reduces animal stress compared to previous models but also offers a reproducible and ethically sound platform for investigating IVDD pathogenesis and testing potential therapeutic interventions.

## Introduction

Globally, chronic low-back pain remains the leading cause of disability, and it is closely linked with intervertebral disc degeneration (IVDD) (GBD 2017 Disease and Injury Incidence and Prevalence Collaborators, 2018). Epidemiological studies have revealed a substantial dose–response relationship between cumulative occupational lumbar load and IVDD, indicating that long-term physical workload is a key risk factor (Seidler et al., 2009). Animal models play an indispensable role in elucidating the pathogenesis of IVDD and evaluating therapeutic strategies, owing to advantages such as easy tissue accessibility, reduced inter-subject variability compared to humans, and the feasibility of conducting *in vivo* experiments (O’Connell, Vresilovic & Elliott, 2007). However, the lack of an animal model that faithfully simulates the chronic loading experienced by the human intervertebral disc during prolonged upright posture has, to some extent, hindered research progress in this field.

Existing animal models of IVDD can be broadly categorized into three types: structural disruption models, mechanical loading models, and chemical disruption models. In mechanical loading models, the continuous loading devices used pose ethical concerns, and chemical disruption models suffer from poor reproducibility due to individual differences in response to chemical agents. Consequently, these two types have not been widely adopted. In contrast, structural disruption models—such as the needle puncture and spinal instability models—have gained popularity due to their relatively short induction time and reproducibility, though their application in early intervention studies remains limited (Jin, Balian & Li, 2018).

The needle puncture model induces IVDD by puncturing the annulus fibrosus with a needle, leading to immediate nucleus pulposus extrusion. This abrupt injury and subsequent inflammatory response differ markedly from the gradual degenerative changes occurring naturally in the nucleus pulposus and annulus fibrosus (Sobajima et al., 2005). Moreover, many studies using this model have focused solely on the structural and biochemical alterations of the disc, neglecting the chronic, naturally progressive course of IVDD (Jin, Balian & Li, 2018). As a result, these models do not fully recapitulate the human degenerative process, thereby limiting their utility in studying IVDD pathogenesis.

In recent years, spinal instability models have attracted increasing attention. Unlike needle puncture, these models do not directly damage the disc; instead, they induce long-term instability of the spinal segment, leading to a slower degeneration that better mimics the clinical pathology of IVDD. In Miyamoto, Yonenobu & Ono (1991) established a reproducible model of cervical spondylosis by dissecting the connections between the posterolateral muscles and the vertebrae and removing the spinous processes and associated ligaments, thereby inducing mechanical instability of the cervical spine. Twelve months postoperatively, marked IVDD and osteophyte formation were observed, demonstrating that long-term mechanical instability can lead to cervical spondylosis. In Qiu, Teo & Zhang (2006) developed a nonlinear three-dimensional finite element model using thoracolumbar human specimens, finding that bilateral facet joint resection increased both the range of motion of the spinal segment and the load borne by the vertebral body. Although this finding provided a theoretical basis for treating spinal instability, the model did not account for the stabilizing role of the paraspinal muscles. In the same year, Zongqiang, Shangli & Zhaomin (2006) reported that resection of the facet joints at L4, L5, and L6 in rabbits induced IVDD. In Guo et al. (2010) revealed in goat cervical spine specimens that even when the paraspinal muscles were preserved, resection of more than 50% of the facet joints resulted in spinal instability. In Ding et al. (2013) established a dynamic and static imbalance model of cervical degeneration in rats by transecting the neck and dorsal muscles and ligaments, thereby simulating the clinical scenario of cervical degeneration resulting from long-term strain and injury to the neck and shoulder muscles. Liu et al. (2014) induced cervical IVDD in rats by resecting the bilateral upper and lower facet joints at the C4/5 and C5/6 levels. In Ao et al. (2019) introduced a novel bipedal standing mouse model by confining mice in a water-containing, bucket-shaped space to force a prolonged bipedal posture, thereby inducing lumbar IVDD.

To date, research on instability models has predominantly focused on larger animals such as sheep, rabbits, and rats, with relatively few studies conducted in mice. Given the advantages of genetic manipulation and the availability of transgenic lines in mice, the development of mouse models of spinal instability would greatly advance IVDD research. Thus, establishing a mouse IVDD model that accurately reflects human degenerative pathology is both scientifically substantial and urgently needed.

Cervical instability alters the normal biomechanical loading environment of the intervertebral disc by increasing shear stress, axial compression, and abnormal rotational motion between vertebral bodies (Miyamoto, Yonenobu & Ono, 1991; Qiu, Teo & Zhang, 2006). These abnormal mechanical forces disrupt the homeostasis between anabolic and catabolic processes in disc cells, leading to extracellular matrix degradation, annulus fibrosus delamination, and nucleus pulposus cell apoptosis (Ao et al., 2019). Over time, the persistent abnormal motion accelerates degenerative changes within both the disc and surrounding structures, thereby establishing a direct biomechanical pathway from cervical instability to intervertebral disc degeneration.

In this study, we present a novel mouse model of cervical instability, achieved by performing bilateral cervical laminectomy and spinous process resection while preserving the facet joints and posterior cervical muscles. This procedure is designed to induce a chronic unstable state in the cervical spine, thereby simulating the pathological process of IVDD driven by instability. We conducted continuous MRI scanning at 12 weeks post-operation to quantitatively track pathological changes and complemented these findings with histological, immunohistochemical, and immunofluorescence analyses.

## Materials & Methods

### Model and study design

A total of 48 male Sprague–Dawley rats (3 months old, weighing 230 ± 20 g) and 48 male C57BL/6 mice (12 weeks old, weighing 20 ± 2 g) were purchased from the Experimental Animal Center of Southern Medical University (Guangzhou). The animals used in this study were healthy, wild-type, free of genetic modifications, and not undergone any previous surgeries. All animals were randomly assigned into four groups based on random numbers generated by Excel (n = 12 per group): sham, 1-month post-surgery, 2-months post-surgery, and 3-months post-surgery, and were housed individually. The sample size was determined based on the required replicates for histological assessments and other analyses. We acknowledge that no formal a priori power analysis was performed, which is a limitation of the current design. Only males were used to eliminate gender-related differences.

In this study, a bilateral cervical laminectomy combined with spinous process resection was performed while preserving the facet joints and posterior cervical muscles. The resulting defect in the laminae induced chronic intervertebral instability of the cervical spine. During routine activities such as feeding and drinking, the mice incurred additional axial and rotational loads on the unstable cervical segments, thereby simulating the pathophysiological process of IVDD observed in humans during upright posture. The detailed surgical procedure was as follows:

For the rats, anesthesia was induced using 4% isoflurane in an oxygen mixture with maintenance at 2% isoflurane (using a small animal anesthesia system, model VIPl00, Matrix, USA). The rat’s head was fixed in a stereotaxic frame, and the posterior neck region was prepped and disinfected. Anatomical landmarks corresponding to the C2 and T2 spinous processes were used for surface localization. A midline incision of approximately seven cm was made between the spinous processes. Following skin incision, the muscles were bluntly separated along natural intermuscular planes and retracted using a small animal retractor. After exposing the laminae from C3 to T1, any soft tissue covering the laminae was carefully removed with a small blade, clearly delineating the bony structure while ensuring that the inner margins of the bilateral facet joints were not damaged. In the surgical groups, a small double-joint rongeur was used to excise the laminae bilaterally from C3 to T1, taking care to avoid injuring the spinal cord. Hemostasis was achieved, and the muscles were preserved and subsequently closed in layers. In the sham group, the skin was incised and the laminae exposed without any bone resection, and the incision was then closed. Postoperatively, all animals received intramuscular injections of penicillin (4 × 10^5^ U/kg) for three consecutive days to prevent infection, and meloxicam (five mg/kg) was administered subcutaneously immediately after surgery to manage postoperative pain. This dosage and route of administration were selected based on established veterinary guidelines for rodent pain management following orthopedic procedures, ensuring effective analgesia while minimizing potential side effects.

The surgical procedure for mice was similar to that for rats, except that microscopic scissors were used to resect the bilateral laminae.

All animals were housed in standard laboratory cages of experimental animal center of Nanfang Hospital under controlled temperature (22 ± 2 °C) and humidity (50 ± 10%) conditions with a 12-hour light/dark cycle. After surgery, each animal was allowed free, unrestricted weight-bearing and activity. Animals were fed a standard laboratory diet and water ad libitum. The housing density was maintained at 3–4 animals per cage to ensure their well-being while allowing for normal social interactions. No specific environmental enrichment items were provided beyond the standard housing setup, as the study design focused on post-surgical recovery and monitoring rather than behavioral assessment.

The primary outcomes measured in this study were the progression and severity of IVDD as assessed by *in vivo* MRI (PharmaScan70/16 US, Bruker, SMU Central Laboratory, Southern Medical University), histologic evaluation (hematoxylin–eosin and Safranin O–fast green staining), immunofluorescence, and immunohistochemistry for collagen Type II and Aggrecan. MRI examinations were performed preoperatively and at 1, 2, and 3 months postoperatively to track continuous IVDD in the 2-month and 3-month groups. Histologic assessment used a modified scoring system where higher scores indicated more severe degeneration. Immunofluorescence and immunohistochemistry analyses quantified the expression levels of key extracellular matrix proteins in the intervertebral discs. Assessors and data analysts were blinded to the group allocations during outcome assessment and data analysis.

All animals were euthanized at 1, 2, and 3 months post-surgery using sodium pentobarbital (150 mg/kg) administered intraperitoneally. This method was chosen for its rapid action and minimization of stress to the animals. Prior to the planned end of the experiment, criteria for early euthanasia included severe neurological deficits (*e.g.*, hindlimb paralysis), inability to access food or water, or weight loss exceeding 20% of baseline body weight. No animals required euthanasia before the scheduled endpoint based on these criteria.

### MRI scanning parameters

In vivo MRI examinations were conducted using a 7.0T PharmaScan 70/16 US system (Bruker, Germany) with a gradient strength of 760 mT/m and a gradient switching rate of 6,840 mT/m/ms. Animals were anesthetized with isoflurane (3% induction, 1.5%–2.0% maintenance) and physiological parameters, including body temperature, heart rate, and respiratory rate, were continuously monitored to maintain stability. T2-weighted images were acquired using a Rapid Acquisition with Refocused Echoes (RARE) sequence. The parameters were: repetition time (TR) = 4,200–5,000 ms; echo time (TE) = 33–52 ms; slice thickness = 0.65 mm; field of view (FOV) = 25.6 × 25.6 mm^2^ or 35 × 35 mm^2^; matrix size = 256 × 256; flip angle = 90°.

### Immunofluorescence

The cervical spine specimens of rats and mice (C2–T2) were collected for histological and immunofluorescence analysis. After fixation in 4% paraformaldehyde for 24–48 h, the intervertebral disc tissues were decalcified using ethylenediaminetetraacetic acid (EDTA) under gentle agitation for at least 8 weeks for rat specimens and a minimum of 2 weeks for mouse specimens. Following decalcification, the tissues were dehydrated, embedded in paraffin, and sectioned at a thickness of four µm. To suppress endogenous peroxidase activity, the sections were treated with 0.3% hydrogen peroxide for 10 min, followed by permeabilization with 0.2% Triton X-100. Non-specific binding was blocked by incubation with 10% goat serum for 30 min. The sections were then incubated overnight at 4 °C with primary antibodies against collagen type II (1:100, Proteintech, 28459-1-AP) and aggrecan (1:100, Proteintech, 13880-1-AP). On the following day, the sections were incubated with a Cy3-conjugated secondary antibody for 1 h at room temperature, and nuclei were counterstained with DAPI. All slides were examined under a ZEISS AXIO Imager D2 microscope (Germany). For each group, fluorescence images were captured under identical exposure settings, and gray values were automatically quantified using the IMAGER D2 system.

### Histologic assessment

After deparaffinization and rehydration, the sections were stained with hematoxylin–eosin (H&E) (Lai et al., 2021) and Safranin O–fast green (Walter et al., 2014) to assess the extent of intervertebral disc degeneration. The degeneration score was calculated using a modified scoring system (Yang et al., 2009), where higher scores indicate more severe degeneration. The observers (KW Tu, XH Dai, K Zhao) were blinded to the group assignments.

### Immunohistochemistry

The mice sections were deparaffinized and rehydrated, followed by antigen retrieval in citric acid buffer (pH 6.0) using a 60 °C water bath for 16 h to unmask antigenic sites and enhance antibody binding. After cooling to room temperature, the sections were incubated with 0.3% hydrogen peroxide solution for 10 min to quench endogenous peroxidase activity, and then blocked with 10% goat serum for 30 min at room temperature to prevent nonspecific binding. Subsequently, the sections were incubated overnight at 4 °C with primary antibodies against collagen type II (1:100, 28459-1-AP; Proteintech, Rosemont, IL, USA) and aggrecan (1:100, 13880-1-AP; Proteintech, Rosemont, IL, USA), followed by incubation with an HRP-conjugated secondary antibody (RGAU011; Proteintech, Rosemont, IL, USA) for 1 h at room temperature. The immunoreactive signals were visualized using 3,3′-diaminobenzidine (DAB) as a chromogenic substrate, and the sections were counterstained with hematoxylin. Finally, the slides were dehydrated, mounted with neutral resin, and observed under an optical microscope (ZEISS AXIO Imager D2, Germany). All investigators (KW Tu, XH Dai, K Zhao) were blinded to group assignments.

### Animal ethics

This study was approved by the Laboratory Animal Care and Use Committee of Nanfang Hospital, Southern Medical University (IACUC-LAC-20230413-017).

### Statistical analyses

**Inclusion criteria:** All rats and mice that survived until the designated experimental endpoint and successfully completed all scheduled procedures (including surgery, MRI scanning, and tissue collection) were included in the statistical analysis.

**Exclusion criteria:** Animals exhibiting severe neurological deficits (*e.g.*, forelimb paralysis), inability to access food or water, or weight loss exceeding 20% of their baseline body weight prior to the experimental endpoint were excluded from the analysis. All statistical analyses were conducted using SPSS 20.0 (SPSS Inc., Armonk, NY, USA). Continuous data are expressed as the mean ± standard deviation (SD), while nonparametric data are presented as the mean ± interquartile range (IQR). For continuous variables, the homogeneity of variance was first assessed. When variances were homogeneous, comparisons between two groups were performed using Student’s *t*-test, and one-way ANOVA followed by Bonferroni *post hoc* tests was applied for comparisons among multiple groups. If the variances were unequal, nonparametric tests were employed—specifically, the Mann–Whitney *U* test for two-group comparisons or the Kruskal–Wallis *H* test for multiple group comparisons. For nonparametric variables such as the disc Pfirrmann grade, the Kruskal–Wallis *H* test was used, with *post hoc* pairwise comparisons conducted using an adjusted α value. A *p*-value of < 0.05 was considered statistically substantial.

## Results

### Establishment of a cervical degeneration model in mice and rats

**Figure 1:**
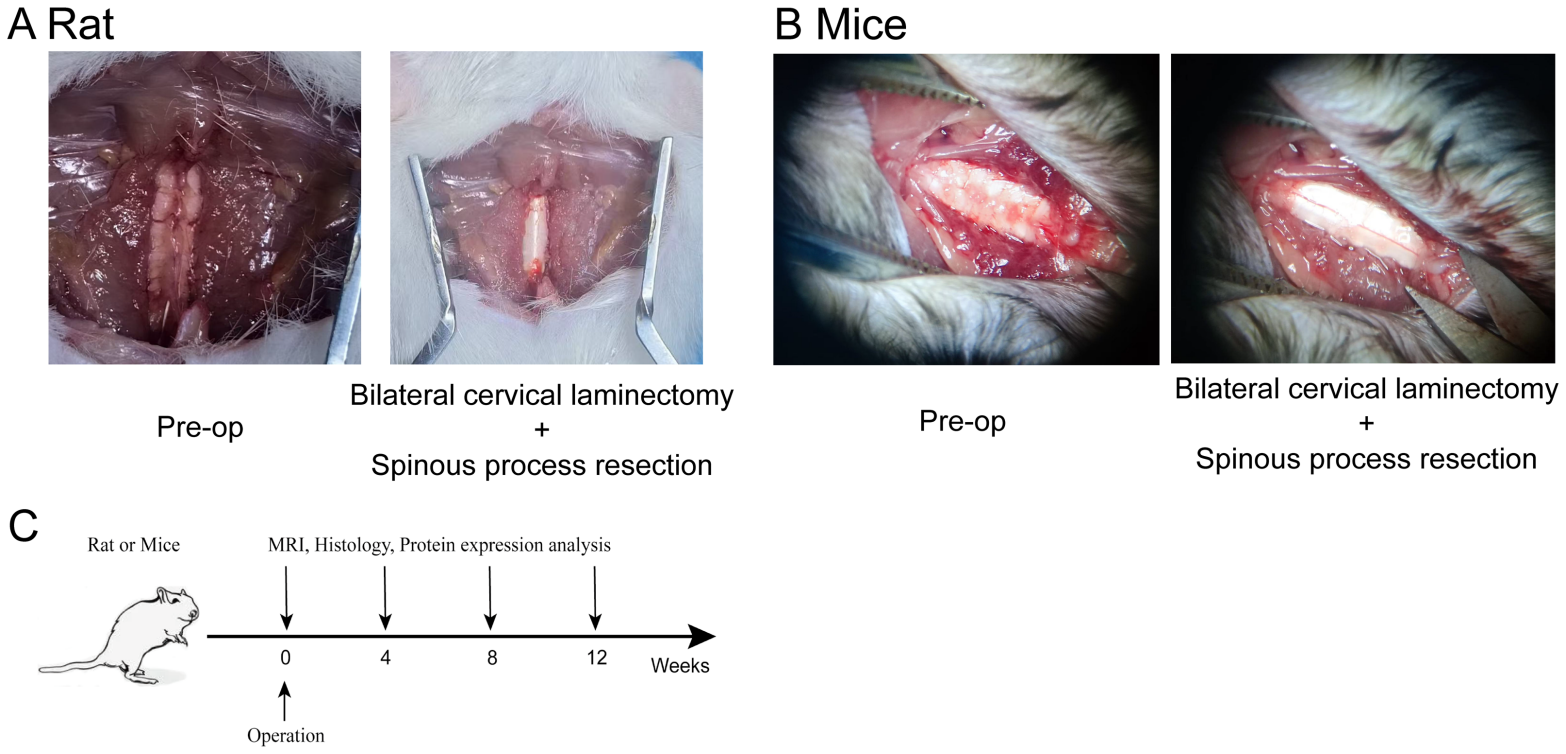
Establishment of the cervical degeneration model in mice and rats. (A) Surgical procedure in rat, demonstrating bilateral cervical laminectomy combined with cervical spinous process resection *via* posterior cervical spine approach. (B) Surgical procedure in mice, showing bilateral cervical laminectomy combined with spinous process resection *via* posterior cervical spine approach. (C) Experimental timeline, indicating the time points for MRI, histological, and protein expression analyses at 4, 8, and 12 weeks post-operation. Photo credit: Kewu Tu and Xiangheng Dai.

To induce cervical degeneration, a surgical approach was implemented in both mice and rats. Figure 1A illustrates the surgical procedure in mice. The cervical skin was incised to fully expose the cervical spine. Using micro-scissors, the laminae of the cervical vertebrae were removed to expose the spinal cord while preserving the facet joints, thereby establishing a model of cervical spinal instability. Similarly, Fig. 1B depicts the surgical procedure in rats, demonstrating the exposure and injury of the cervical intervertebral disc. The experimental timeline is shown in Fig. 1C, indicating that MRI scans, histological analysis, and protein expression assessments were conducted at 4, 8, and 12 weeks post-operation.

### MRI analysis of cervical degeneration in mice and rats

**Figure 2:**
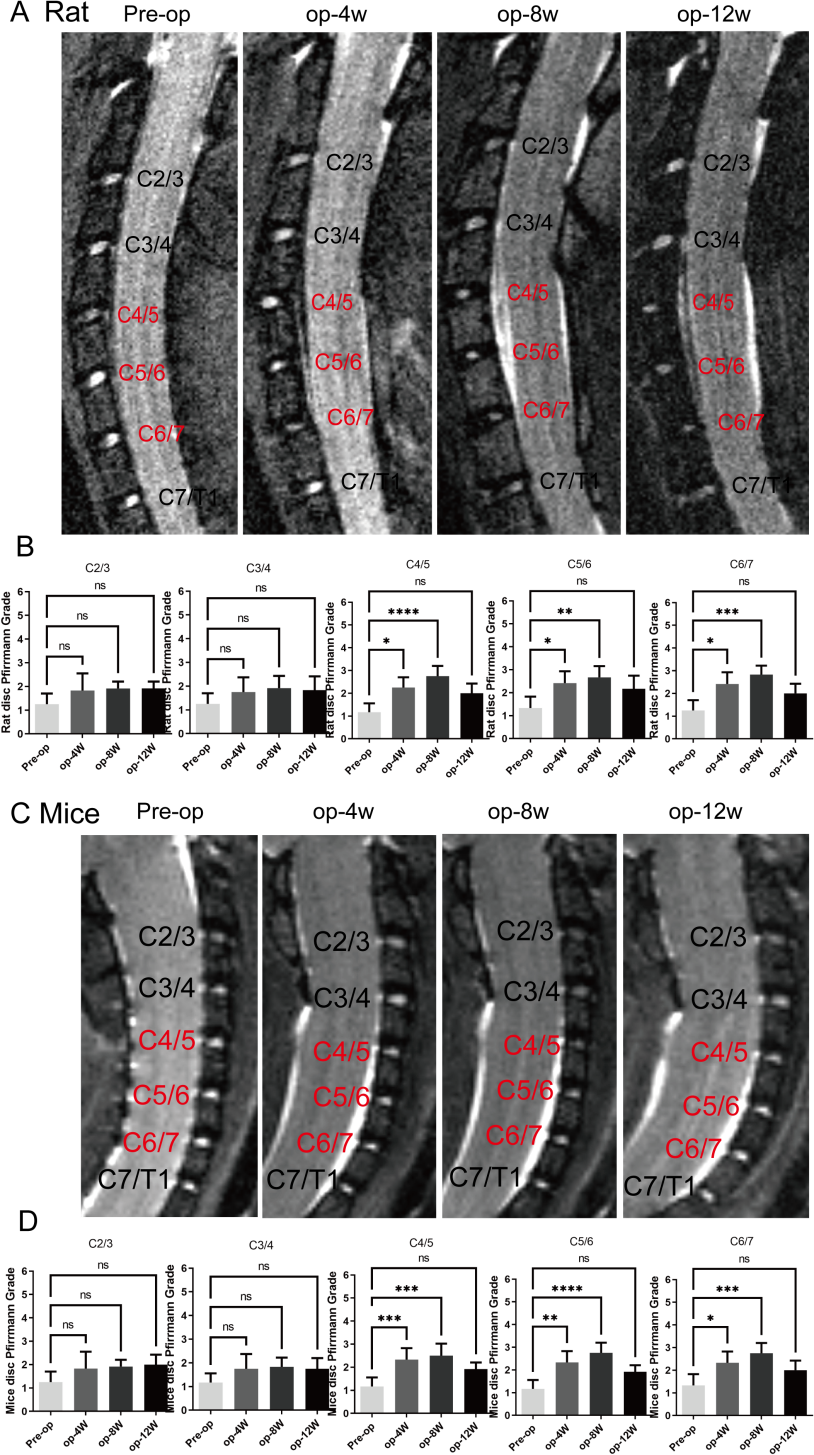
MRI evaluation of cervical degeneration in mice and rats. (A) Representative MRI images of rats at pre-operation (Pre-op), 4 weeks (op-4 w), 8 weeks (op-8 w), and 12 weeks (op-12 w) post-surgery, showing progressive disc degeneration at C4/5, C5/6, and C6/7. (B) Pfirmann scores of rats at different cervical segments, demonstrating significant degeneration at 1 and 2 months, with no significant differences at 3 months. (*n* = 12) (C) Representative MRI images of mice at pre-operation, 4 weeks, 8 weeks, and 12 weeks post-surgery, showing similar degeneration patterns as in rats. (D) Pfirmann scores of mice at different cervical segments, revealing statistical significance at 1 and 2 months, but no significant differences at 3 months. (*n* = 12). ns: no statistical significance, * *P* < 0.05, **P < 0.01, ***P < 0.001, ****P < 0.0001.

MRI was performed to evaluate the progression of cervical degeneration over time. As shown in Figs. 2A and 2C, MRI images of both mice and rats revealed progressive cervical disc degeneration at C4-5, C5-6, and C6-7, with the most pronounced degeneration observed at 4 and 8 weeks post-operation. Quantitative assessment using the Pfirmann grading system (Figs. 2B and 2D) validated a substantial increase in degeneration at C4-5, C5-6, and C6-7 at 1 and 2 months (*n* = 12) (*p* < 0.05), while no substantial differences were detected at 3 months, indicating partial recovery in both models.

### Histological, immunofluorescence changes and western blot analysis in the rat cervical intervertebral discs

**Figure 3:**
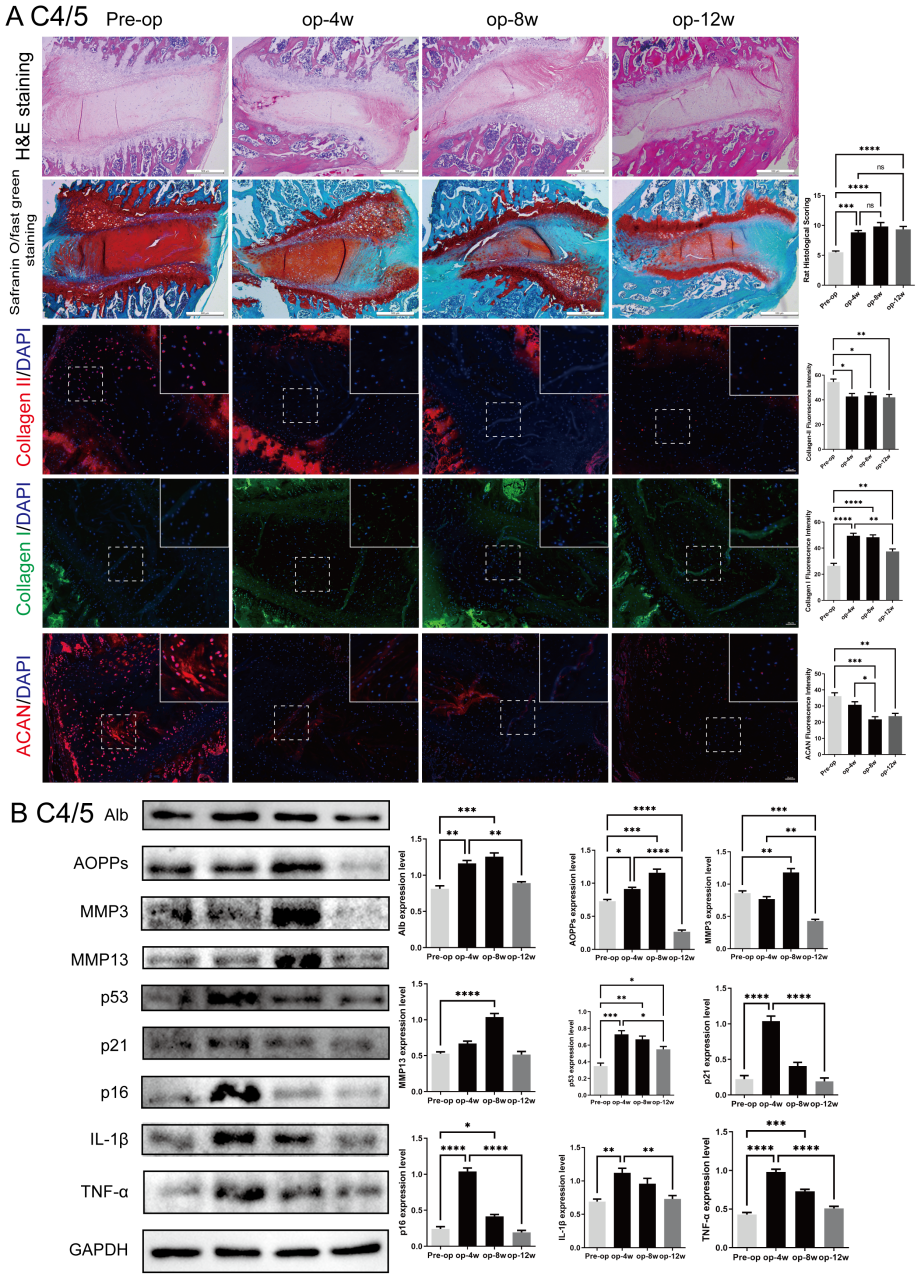
Histological, immunofluorescent, and protein expression changes in the rat C4/5 cervical disc of rats post-surgery. (A) HE and Safranin O-fast green staining of C4/5 discs at 0, 4, 8, and 12 weeks post-surgery showing progressive disc degeneration, including decreased NP cellularity, blurred NP-AF boundaries, and endplate disruption. Immunofluorescence staining showed decreasing expression of Col-II and ACAN, with increased Col-I expression over time. (B) Western blot analysis of disc tissues revealed elevated expression of Alb, AOPPs, MMP3, MMP13, p53, p21, p16, IL-1β, and TNF-α at 4 and 8 weeks, with partial decline at 12 weeks. Scale bars: 5 00 µm (histology); 2 0 µm (immunofluorescence). ns: no statistical significance, * *P* < 0.05, **P < 0.01, ***P < 0.001, ****P < 0.0001.

To evaluate the progression of cervical disc degeneration, histological and molecular changes were examined in the C4/5 segment of rat intervertebral discs following surgical intervention (Fig. 3).

As shown in Fig. 3A, hematoxylin-eosin (H&E) and Safranin O-fast green staining revealed a time-dependent worsening of IVDD at 4, 8, and 12 weeks post-surgery. Characteristic features included a gradual loss of nucleus pulposus (NP) cells, unclear boundaries between the NP and annulus fibrosus (AF), thinning of the cartilage endplate, and structural disruption. These degenerative changes were most pronounced at 8 weeks, although mild structural recovery was observed in adjacent segments such as C2/3 and C3/4 at 12 weeks.

Immunofluorescence staining showed a progressive decrease in collagen type II (Col-II) and aggrecan (ACAN), key components of the extracellular matrix (ECM), with increasing post-operative time. Conversely, expression of collagen type I (Col-I) increased over time, indicating a shift toward fibrosis. These findings support a sustained degenerative process at the molecular level.

Western blot analysis (Fig. 3B) of C4/5 disc tissue confirmed these observations. Expression levels of oxidative stress markers (albumin (Alb) and advanced oxidation protein products (AOPPs)), matrix metalloproteinases (MMP3 and MMP13), senescence markers (p53, p21, p16), and inflammatory cytokines (IL-1β, TNF-α) were elevated at 4 and 8 weeks, with a partial decline by 12 weeks. These results validate the rat model as an effective model of progressive cervical disc degeneration, accompanied by oxidative stress, ECM catabolism, cellular senescence, and inflammation. Comparable trends were observed in other cervical segments (C2/3, C3/4, C5/6, and C6/7), as shown in Figs. S1–S4.

### Histological evaluation and immunohistochemistry of cervical disc degeneration in mice

**Figure 4:**
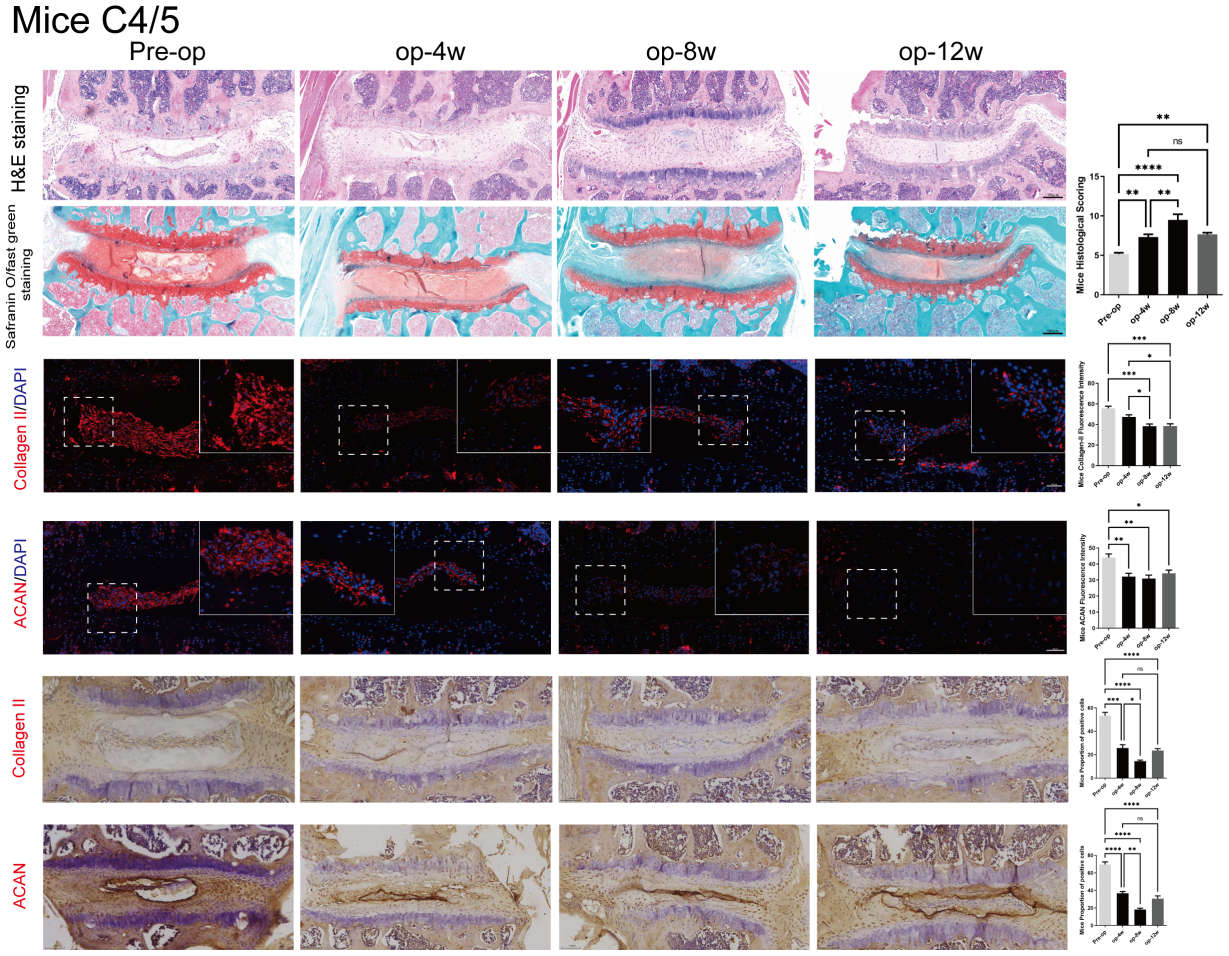
Histological, Immunofluorescence and Immunohistochemistry assessment of mice C4/5 cervical discs in mice post-surgery. HE (top row) and Safranin O staining (second row) demonstrate progressive degenerative changes from 0 to 12 weeks. Immunofluorescence (3–4 rows) and Immunohistochemistry (5–6 rows) images show declining expression of ACAN and Col-II over time. Quantitative analysis of ACAN-positive cells and Col-II fluorescence intensity (*n* = 3), revealing significant reductions at 4 and 8 weeks. Scale bars: 1 00 μm (H istology and Immunohistochemistry); 5 0 μm (I mmunofluorescence). ns: no statistical significance, * *P* < 0.05, **P < 0.01, ***P < 0.001, ****P < 0.0001.

Figure 4 illustrates the degenerative changes observed in the C4/5 segment of mouse intervertebral discs.

Histological analysis using H&E and Safranin O staining demonstrated time-dependent degeneration, similar to the rat model. This was characterized by decreased NP cellularity, blurred NP–AF boundaries, and thinning and rupture of the endplate. These pathological changes were progressively aggravated from 4 to 12 weeks post-surgery. Similar degenerative patterns were also observed in adjacent cervical segments (C2/3, C3/4, C5/6, and C6/7), as shown in Fig. S5.

Immunofluorescence staining and Immunohistochemistry indicated that both ACAN and Col-II expression decreased significantly over time, reflecting ongoing matrix loss. Quantitative analysis revealed significantly reduced ACAN-positive cell ratios and Col-II fluorescence intensity at 4 and 8 weeks (*p* < 0.05), with partial recovery at 12 weeks. The expression patterns are consistent with the histological observations, further validating the mouse model for studying cervical disc degeneration.

## Discussion

Intervertebral disc degeneration (IVDD) represents a multifactorial pathological process influenced by genetic, mechanical, inflammatory, and biochemical factors (GBD 2017 Disease and Injury Incidence and Prevalence Collaborators, 2018; Seidler et al., 2009; O’Connell, Vresilovic & Elliott, 2007). Chronic mechanical instability of the cervical spine has been increasingly recognized as a critical contributor to IVDD progression (Jin, Balian & Li, 2018; Sobajima et al., 2005; Miyamoto, Yonenobu & Ono, 1991). The present study aimed to establish a reproducible and physiologically relevant animal model that mimics the chronic degenerative changes caused by cervical instability. Using bilateral cervical laminectomy and spinous process resection while preserving the facet joints and posterior cervical muscles, we successfully induced progressive cervical IVDD in both rats and mice. MRI, histological, and molecular analyses consistently demonstrated time-dependent degenerative changes, confirming that this model effectively simulates cervical disc degeneration associated with chronic instability.

Our findings align with previous studies demonstrating that altered spinal biomechanics accelerate disc degeneration through increased shear stress, compression, and abnormal motion between vertebral segments (Qiu, Teo & Zhang, 2006; Zongqiang, Shangli & Zhaomin, 2006). Similar to reports by Miyamoto, Yonenobu & Ono (1991) and Zongqiang, Shangli & Zhaomin (2006), we observed progressive structural disruption and extracellular matrix degradation. However, unlike the needle puncture model—which induces acute injury and rapid degeneration (Jin, Balian & Li, 2018; Sobajima et al., 2005)—our model replicates the chronic and gradual degenerative process more consistent with human pathology. The partial recovery observed at 12 weeks likely reflects compensatory remodeling by adjacent segments and surrounding tissues, consistent with previous evidence that fibrosis and adaptive remodeling occur in response to chronic instability rather than true biological regeneration (Ashinsky et al., 2021; Kim et al., 2015).

Compared with other existing models, such as the facet joint resection model (Liu et al., 2014) and the bipedal standing mouse model (Ao et al., 2019), our approach provides distinct biomechanical, ethical, and technical advantages. Both large and small rodents are quadrupedal animals, and the types of loads borne by their lumbar spine differ substantially from those experienced by humans in an upright posture. The spinal instability models created by resecting the posterior elements of the lumbar spine fail to accurately reproduce the human intervertebral disc degeneration (IVDD) process, which primarily develops under chronic vertical and rotational loading. Under standard laboratory housing conditions, both rats and mice must elevate their heads to access food and water. As a result, their cervical spine endures greater axial compression and rotational forces than the lumbar spine. Based on these biomechanical characteristics, we hypothesize that a cervical spine instability model in rodents may more accurately mimic the chronic axial and torsional stresses that contribute to IVDD in humans during prolonged upright posture.

The facet joint resection model (Liu et al., 2014), although capable of inducing disc degeneration through joint destabilization, involves extensive tissue dissection due to the close proximity of the facet joints to the spinal nerve roots. Bilateral resection of these joints requires a high degree of microsurgical precision, and even minor procedural errors—such as incomplete resection or accidental nerve root injury—can lead to postoperative neurological deficits, secondary inflammation, or even death. Consequently, this model carries an increased risk of complications, variability in outcomes, and reduced reproducibility across operators.

The Bipedal Standing Mouse Model (Ao et al., 2019), on the other hand, simulates chronic axial loading through behavioral manipulation rather than surgery. In this model, mice are placed in a water-containing cylindrical container for six hours daily over a 10-week period, forcing them to stand upright to avoid drowning. Although this setup can induce lumbar IVDD, it is associated with considerable stress and ethical concerns. Continuous exposure to a fear-inducing environment can lead to elevated stress hormone levels, behavioral depression, and increased mortality. Moreover, during colder months, the lower water temperature further increases the risk of death. From a biomechanical standpoint, this model applies generalized load across the entire vertebral column, potentially affecting not only the lumbar discs but also the thoracic and cervical segments. This lack of regional specificity limits its suitability for targeted investigations of cervical degeneration.

In contrast, our model induces cervical instability by performing bilateral cervical laminectomy and spinous process resection while preserving the facet joints and posterior cervical muscles. This procedure avoids direct nerve root manipulation, minimizing neurological risk and postoperative complications. The animals remain freely mobile after surgery, requiring no specialized monitoring or modification to the feeding apparatus. During normal activities such as feeding and drinking, they naturally raise their heads and assume semi-upright postures, producing repetitive axial compression and rotational forces on the cervical spine. The absence of the lamina creates a chronic structural instability that amplifies shear stress between adjacent vertebrae, leading to continuous micro-motion, muscle strain, facet joint overload, and progressive intervertebral disc degeneration. Thus, this model closely replicates the biomechanical and pathological conditions observed in human cervical IVDD caused by chronic instability, muscle fatigue, and facet joint degeneration. Furthermore, the intervention is anatomically confined to the cervical region, minimizing off-target effects on the thoracic or lumbar spine, while also ensuring animal welfare and reproducibility.

The use of *in vivo* MRI enabled continuous monitoring of disc degeneration over time, providing longitudinal insights into the progression of structural and biochemical alterations. Histological and immunofluorescence analyses further confirmed extracellular matrix degradation and incomplete repair, consistent with molecular markers of oxidative stress, inflammation, and senescence. Together, these findings highlight that chronic cervical instability can induce sustained degenerative remodeling at both the tissue and molecular levels. Moreover, the typical duration of degeneration in this model—approximately two months—balances experimental feasibility with sufficient time for chronic pathological changes, making it well suited for studying therapeutic interventions and mechanistic pathways of IVDD. While our cervical instability model provides valuable insights into IVDD, there are several limitations. First, species-specific differences between rodents and humans, such as variations in spinal structure and mechanical loading, may limit the direct translation of results to human conditions. Second, the three-month duration of the model may not capture the full range of degenerative changes seen in long-term human IVDD. Third, while *in vivo* MRI offers continuous monitoring, its resolution may not detect fine microstructural changes, and it cannot fully capture biochemical alterations within the disc. Fourth, the model does not include behavioral or pain-related assessments, which slightly weakens its translational impact. We acknowledge this limitation and plan to incorporate behavioral analyses such as gait assessment, grip strength, and mechanical allodynia testing in future studies to better evaluate functional outcomes. Fifth, the model does not yet explore neural involvement or mobility impairment, both of which would further enhance clinical relevance. Although these were beyond the scope of the current study, they represent important future directions to strengthen the translational value of this model. Sixth, the study endpoint of 12 weeks was selected to capture intermediate stages of degeneration and compensatory responses within a feasible experimental timeframe. We acknowledge that long-term follow-up is necessary to assess advanced degenerative features, and future work will extend the model duration to evaluate later-stage degeneration and functional outcomes. Seventh, although we did not assess cervical alignment or mechanical instability with radiographs or CT scans in this study, we agree that these modalities would provide valuable confirmation of structural alterations. And we plan to include X-ray or micro-CT assessments in future studies to strengthen mechanical validation of the model. Lastly, while the model avoids the risks of nerve root manipulation, the surgical procedure requires precision, and variability in surgical outcomes may affect reproducibility. These limitations should be considered when interpreting the results, and addressing them in future studies could further improve the model’s applicability.

In summary, this study establishes a novel and ethically feasible model of cervical spine instability that reliably induces chronic, progressive IVDD in both rats and mice. The model reproduces the biomechanical and molecular features of human cervical degeneration and provides a practical platform for investigating underlying mechanisms and testing therapeutic interventions.

## Conclusions

The cervical instability model presented here effectively simulates the chronic IVDD seen in humans, providing a valuable tool for studying the pathophysiology of IVDD and evaluating potential treatments. While the model has some limitations, such as species-specific differences and the inability to fully capture long-term degeneration, it offers a controlled and reproducible method to investigate cervical spine instability without excessive stress on the animals. Future studies can refine this model to enhance its applicability and further explore therapeutic interventions for IVDD.

## Supplemental Information

10.7717/peerj.20465/supp-1 Supplemental Information 1 ARRIVE 2.0 Checklist

10.7717/peerj.20465/supp-2 Supplemental Information 2 Histological and i mmunofluorescence changes of C2/3 segment of cervical intervertebral disc in rats (A) H&E staining, safranin O-fast green staining, Col II/DAPI staining, Col I/DAPI staining, and ACAN/DAPI staining showed histological changes before surgery (Pre op), 4 weeks after surgery (op-4w), 8 weeks after surgery (op-8w), and 12 weeks after surgery (op-12w). The bar chart on the right displays the corresponding quantitative analysis results. (B) Protein expression analysis of C2/3 segments. The Western blot results showed the expression levels of AOPPs, MMP3, MMP13, p53, p21, p16, IL-1β, and TNF-α, with GAPDH as the internal reference. The bar chart displays the changes in expression levels at different time points. Scale bars: 500 μm (histology); 20 μm (immunofluorescence). ns: no statistical significance, * *P* < 0.05, **P < 0.01, ***P < 0.001, ****P < 0.0001.

10.7717/peerj.20465/supp-3 Supplemental Information 3 Histological and immunofluorescence changes of C 3/4 segment of cervical intervertebral disc in rats (A) H&E staining, safranin O-fast green staining, Col II/DAPI staining, Col I/DAPI staining, and ACAN/DAPI staining showed histological changes before surgery (Pre op), 4 weeks after surgery (op-4w), 8 weeks after surgery (op-8w), and 12 weeks after surgery (op-12w). The bar chart on the right displays the corresponding quantitative analysis results. (B) Protein expression analysis of C 3/4 segments. The Western blot results showed the expression levels of AOPPs, MMP3, MMP13, p53, p21, p16, IL-1β, and TNF-α, with GAPDH as the internal reference. The bar chart displays the changes in expression levels at different time points. Scale bars: 500 μm (histology); 20 μm (immunofluorescence). ns: no statistical significance, * *P* < 0.05, **P < 0.01, ***P < 0.001, ****P < 0.0001.

10.7717/peerj.20465/supp-4 Supplemental Information 4 Histological and immunofluorescence changes of C 5/6 segment of cervical intervertebral disc in rats (A) H&E staining, safranin O-fast green staining, Col II/DAPI staining, Col I/DAPI staining, and ACAN/DAPI staining showed histological changes before surgery (Pre op), 4 weeks after surgery (op-4w), 8 weeks after surgery (op-8w), and 12 weeks after surgery (op-12w). The bar chart on the right displays the corresponding quantitative analysis results. (B) Protein expression analysis of C 5/6 segments. The Western blot results showed the expression levels of AOPPs, MMP3, MMP13, p53, p21, p16, IL-1β, and TNF-α, with GAPDH as the internal reference. The bar chart displays the changes in expression levels at different time points. Scale bars: 500 μm (histology); 20 μm (immunofluorescence). ns: no statistical significance, * *P* < 0.05, **P < 0.01, ***P < 0.001, ****P < 0.0001.

10.7717/peerj.20465/supp-5 Supplemental Information 5 Histological and immunofluorescence changes of C 6/7 segment of cervical intervertebral disc in rats (A) H&E staining, safranin O-fast green staining, Col II/DAPI staining, Col I/DAPI staining, and ACAN/DAPI staining showed histological changes before surgery (Pre op), 4 weeks after surgery (op-4w), 8 weeks after surgery (op-8w), and 12 weeks after surgery (op-12w). The bar chart on the right displays the corresponding quantitative analysis results. (B) Protein expression analysis of C 6/7 segments. The Western blot results showed the expression levels of AOPPs, MMP3, MMP13, p53, p21, p16, IL-1β, and TNF-α, with GAPDH as the internal reference. The bar chart displays the changes in expression levels at different time points. Scale bars: 500 μm (histology); 20 μm (immunofluorescence). ns: no statistical significance, * *P* < 0.05, **P < 0.01, ***P < 0.001, ****P < 0.0001.

10.7717/peerj.20465/supp-6 Supplemental Information 6 Histological changes of C2/3, C3/4, C5/6, and C6/7 segments of cervical intervertebral discs in mice (A–D) H&E staining and Safranin O-fast green staining of cervical spine segments C2/3 (A), C3/4 (B), C5/6 (C), and C6/7 (D) showed histological changes before surgery (Pre op) and 4, 8, and 12 weeks after surgery (op-4w, op-8w, op-12w). Bar charts on the right display corresponding quantitative analysis results. Scale bars: 1 00 μm (histology). ns: no statistical significance, * *P* < 0.05, **P < 0.01, ***P < 0.001, ****P < 0.0001.

## Additional Information and Declarations

### Data Availability

The following information was supplied regarding data availability: The raw data is available at Figshare: Dai, Xiangheng (2025). Chronic Cervical Instability in Mice and Rat A Reproducible Model to Simulate Human Intervertebral Disc Degeneration–raw data. figshare. Dataset. https://doi.org/10.6084/m9.figshare.28560470.v4.
