# Supplementary figures and images for: Chronic cervical instability in mice and rats: a reproducible model to simulate human intervertebral disc degeneration
Source: PeerJ. 2025 Dec 8;13:e20465. doi: 10.7717/peerj.20465 (PMC12697298; doi:10.7717/peerj.20465)

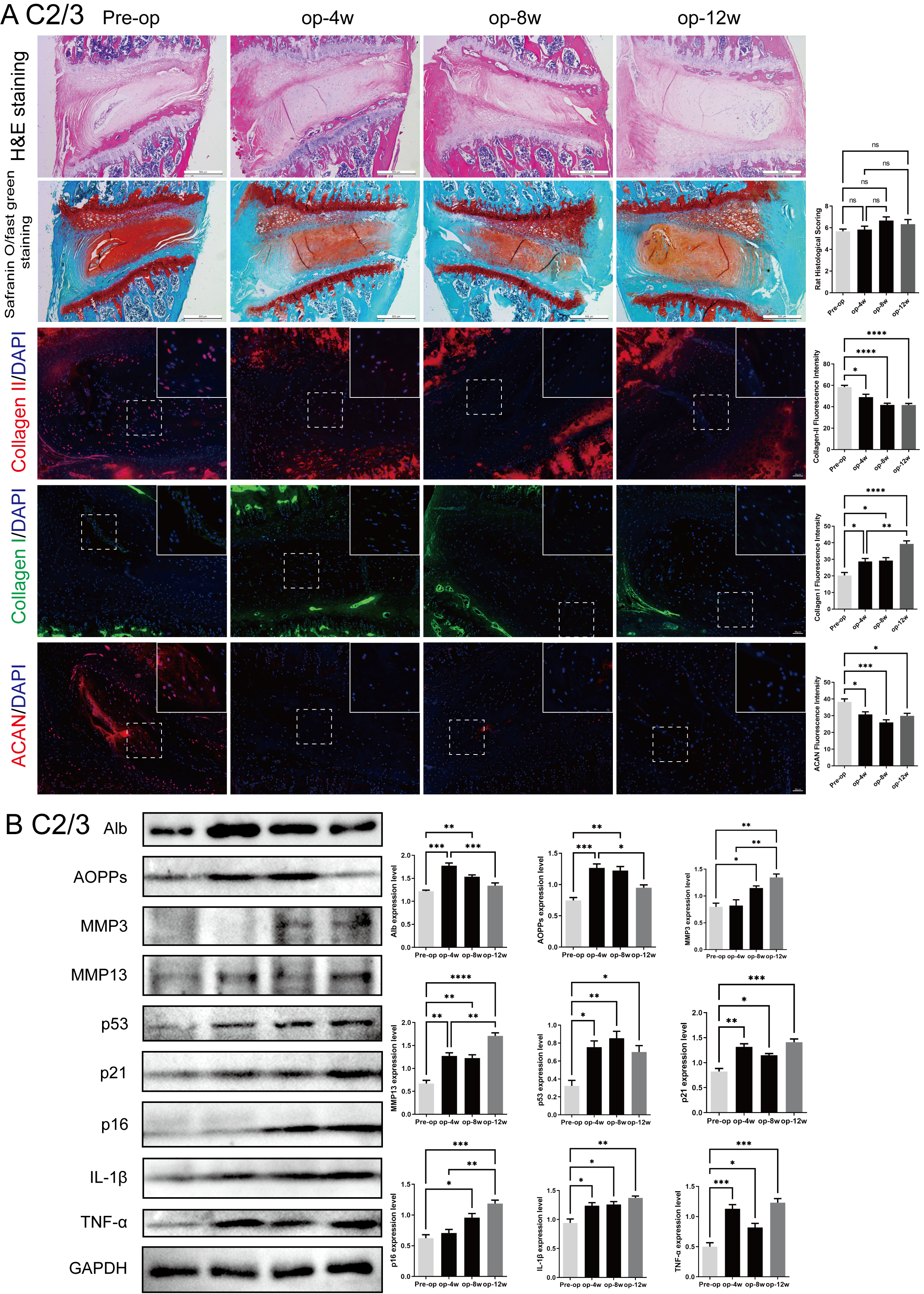

Supplement: Supplemental Information 2 — (A) H&E staining, safranin O-fast green staining, Col II/DAPI staining, Col I/DAPI staining, and ACAN/DAPI staining showed histological changes before surgery (Pre op), 4 weeks after surgery (op-4w), 8 weeks after surgery (op-8w), and 12 weeks after surgery (op-12w). The bar chart on the right displays the corresponding quantitative analysis results. (B) Protein expression analysis of C2/3 segments. The Western blot results showed the expression levels of AOPPs, MMP3, MMP13, p53, p21, p16, IL-1β, and TNF-α, with GAPDH as the internal reference. The bar chart displays the changes in expression levels at different time points. Scale bars: 500 μm (histology); 20 μm (immunofluorescence). ns: no statistical significance, * P < 0.05, **P < 0.01, ***P < 0.001, ****P < 0.0001. [file peerj-13-20465-s002.png]

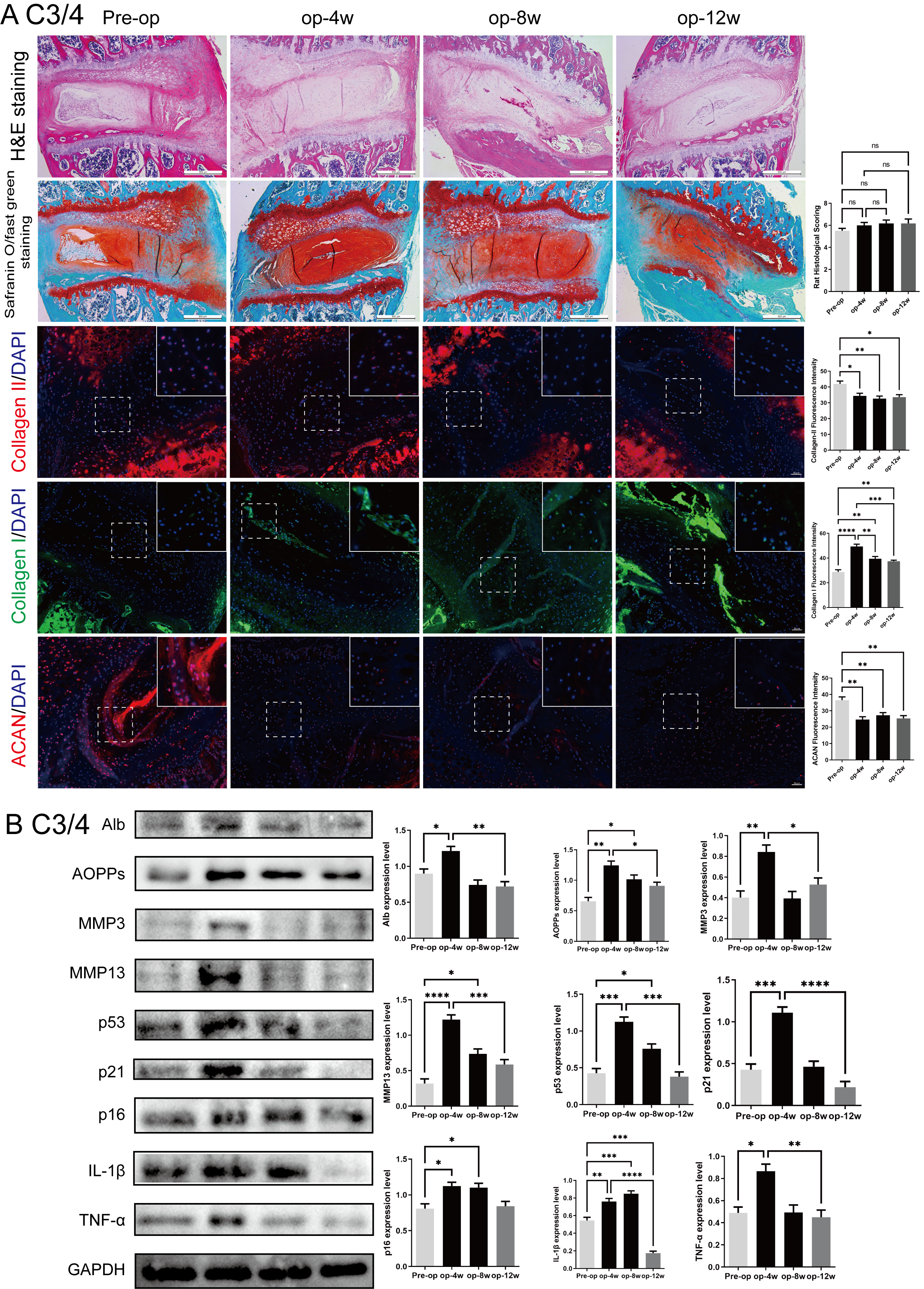

Supplement: Supplemental Information 3 — (A) H&E staining, safranin O-fast green staining, Col II/DAPI staining, Col I/DAPI staining, and ACAN/DAPI staining showed histological changes before surgery (Pre op), 4 weeks after surgery (op-4w), 8 weeks after surgery (op-8w), and 12 weeks after surgery (op-12w). The bar chart on the right displays the corresponding quantitative analysis results. (B) Protein expression analysis of C 3/4 segments. The Western blot results showed the expression levels of AOPPs, MMP3, MMP13, p53, p21, p16, IL-1β, and TNF-α, with GAPDH as the internal reference. The bar chart displays the changes in expression levels at different time points. Scale bars: 500 μm (histology); 20 μm (immunofluorescence). ns: no statistical significance, * P < 0.05, **P < 0.01, ***P < 0.001, ****P < 0.0001. [file peerj-13-20465-s003.png]

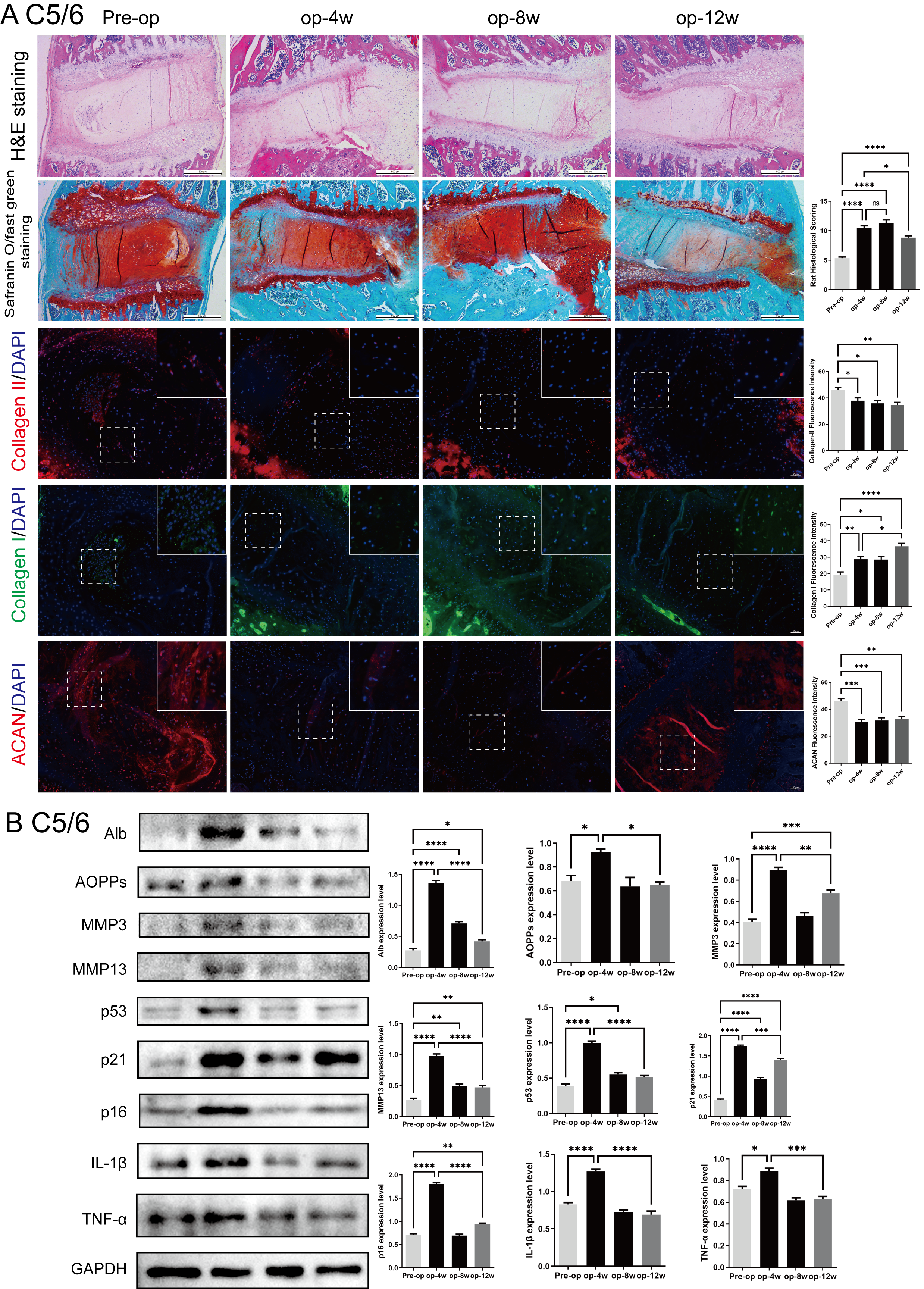

Supplement: Supplemental Information 4 — (A) H&E staining, safranin O-fast green staining, Col II/DAPI staining, Col I/DAPI staining, and ACAN/DAPI staining showed histological changes before surgery (Pre op), 4 weeks after surgery (op-4w), 8 weeks after surgery (op-8w), and 12 weeks after surgery (op-12w). The bar chart on the right displays the corresponding quantitative analysis results. (B) Protein expression analysis of C 5/6 segments. The Western blot results showed the expression levels of AOPPs, MMP3, MMP13, p53, p21, p16, IL-1β, and TNF-α, with GAPDH as the internal reference. The bar chart displays the changes in expression levels at different time points. Scale bars: 500 μm (histology); 20 μm (immunofluorescence). ns: no statistical significance, * P < 0.05, **P < 0.01, ***P < 0.001, ****P < 0.0001. [file peerj-13-20465-s004.png]

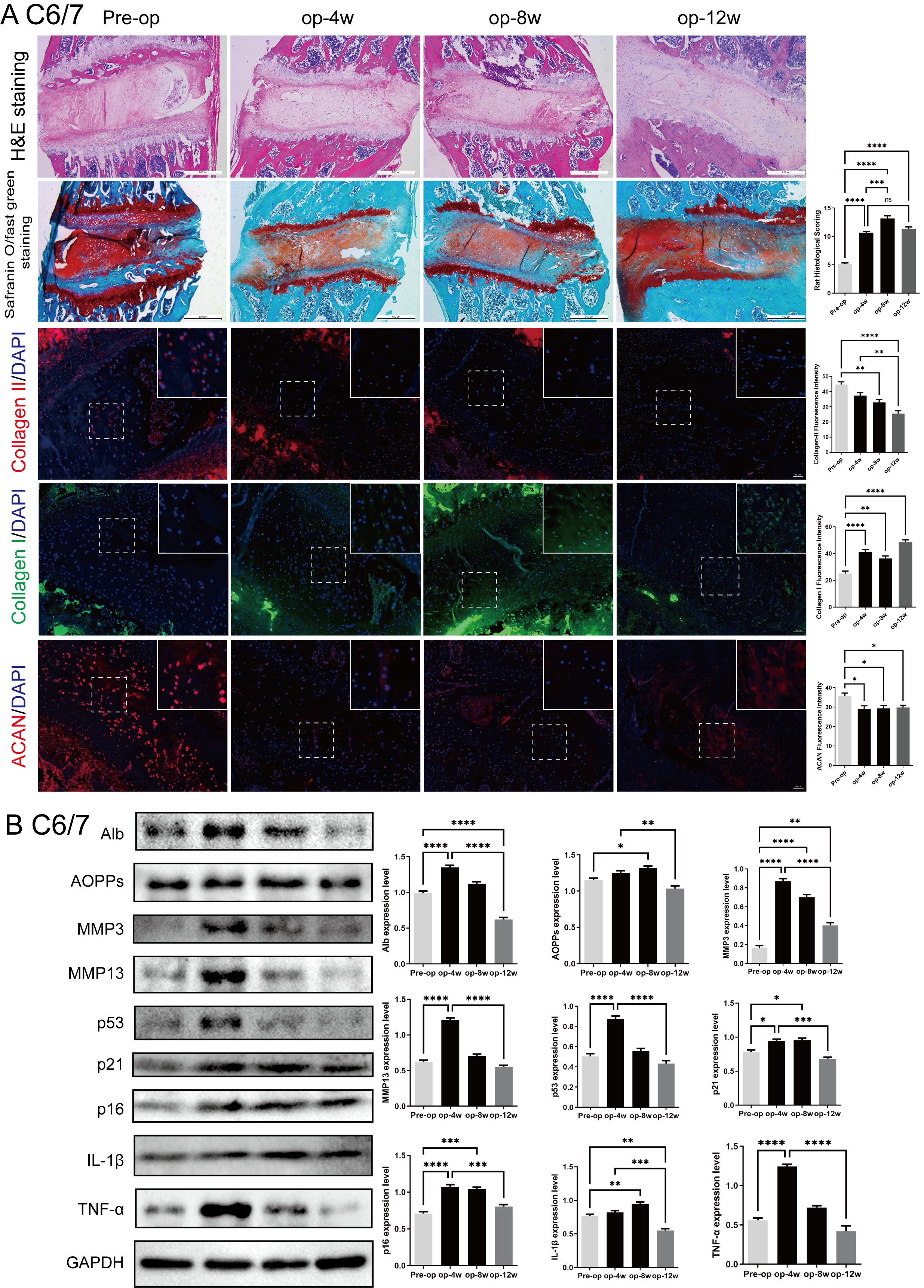

Supplement: Supplemental Information 5 — (A) H&E staining, safranin O-fast green staining, Col II/DAPI staining, Col I/DAPI staining, and ACAN/DAPI staining showed histological changes before surgery (Pre op), 4 weeks after surgery (op-4w), 8 weeks after surgery (op-8w), and 12 weeks after surgery (op-12w). The bar chart on the right displays the corresponding quantitative analysis results. (B) Protein expression analysis of C 6/7 segments. The Western blot results showed the expression levels of AOPPs, MMP3, MMP13, p53, p21, p16, IL-1β, and TNF-α, with GAPDH as the internal reference. The bar chart displays the changes in expression levels at different time points. Scale bars: 500 μm (histology); 20 μm (immunofluorescence). ns: no statistical significance, * P < 0.05, **P < 0.01, ***P < 0.001, ****P < 0.0001. [file peerj-13-20465-s005.png]

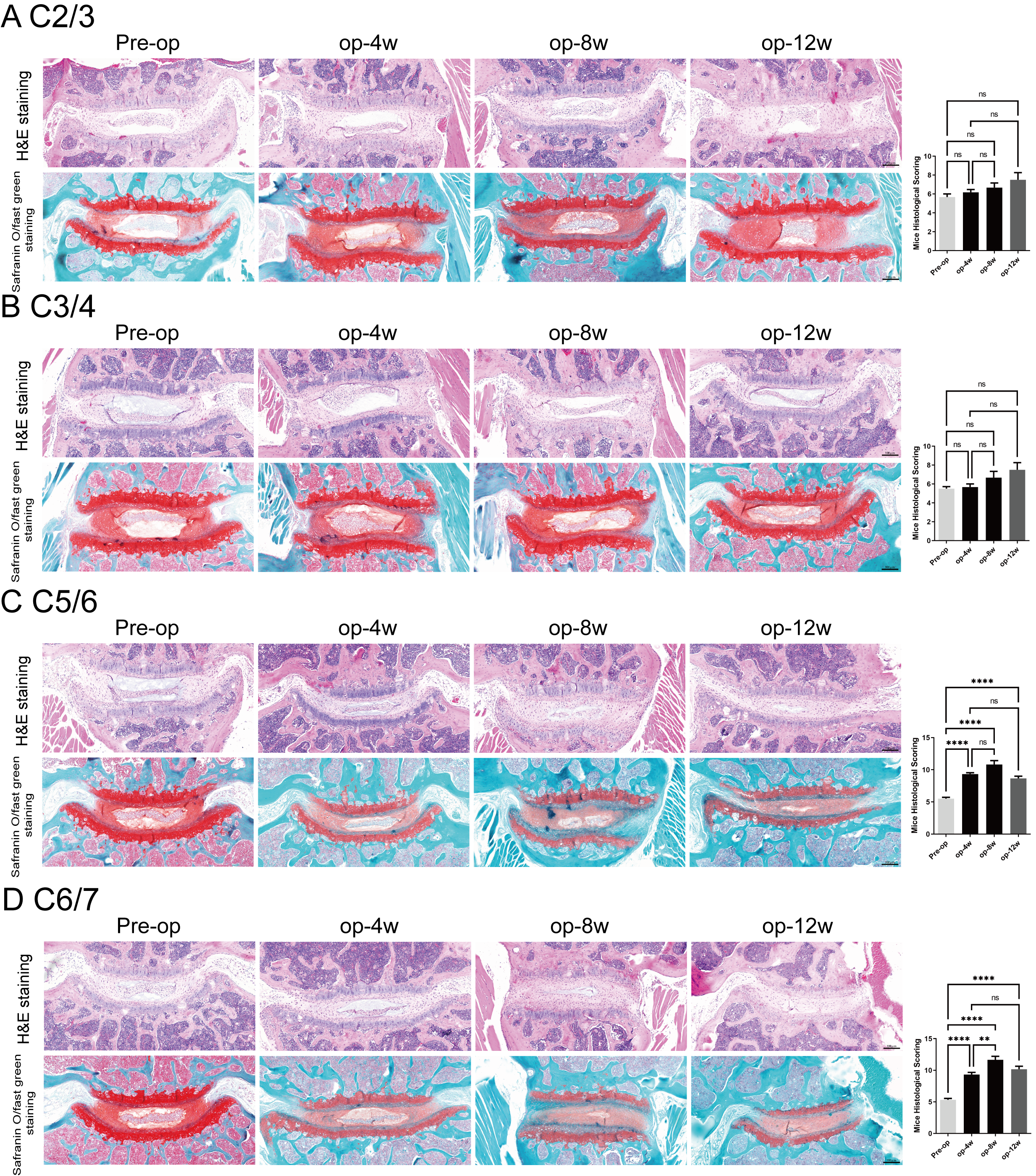

Supplement: Supplemental Information 6 — (A–D) H&E staining and Safranin O-fast green staining of cervical spine segments C2/3 (A), C3/4 (B), C5/6 (C), and C6/7 (D) showed histological changes before surgery (Pre op) and 4, 8, and 12 weeks after surgery (op-4w, op-8w, op-12w). Bar charts on the right display corresponding quantitative analysis results. Scale bars: 1 00 μm (histology). ns: no statistical significance, * P < 0.05, **P < 0.01, ***P < 0.001, ****P < 0.0001. [file peerj-13-20465-s006.png]
